# Phase-Retrieved Tomography enables Mesoscopic imaging of Opaque Tumor Spheroids

**DOI:** 10.1038/s41598-017-12193-x

**Published:** 2017-09-19

**Authors:** Daniele Ancora, Diego Di Battista, Georgia Giasafaki, Stylianos E. Psycharakis, Evangelos Liapis, Jorge Ripoll, Giannis Zacharakis

**Affiliations:** 1 Institute of Electronic Structure and Laser, Foundation for Research and Technology Hellas, GR-70013 Heraklion, Greece; 2 Department of Materials Science and Technology, University of Crete, GR-71003 Heraklion, Greece; 3 School of Medicine, University of Crete, GR-71003 Heraklion, Greece; 4 Department of Bioengineering and Aerospace Engineering, Universidad Carlos III de Madrid, 28911 Madrid, Spain; 5 Instituto de Investigación Sanitaria del Hospital Gregorio Marañón, 28007 Madrid, Spain

## Abstract

We present a new Phase-Retrieved Tomography (PRT) method to radically improve mesoscopic imaging at regimes beyond one transport mean-free-path and achieve high resolution, uniformly throughout the volume of opaque samples. The method exploits multi-view acquisition in a hybrid Selective Plane Illumination Microscope (SPIM) and Optical Projection Tomography (OPT) setup and a three-dimensional Gerchberg-Saxton phase-retrieval algorithm applied in 3D through the autocorrelation sinogram. We have successfully applied this innovative protocol to image optically dense 3D cell cultures in the form of tumor spheroids, highly versatile models to study cancer behavior and response to chemotherapy. We have thus achieved a significant improvement of resolution in depths not yet accessible with the currently used methods in SPIM/OPT, while overcoming all registration and alignment problems inherent to these techniques.

## Introduction

The use of light for visualizing biological processes in living organisms has been a foundational paradigm for biology for almost four centuries, since the invention of the microscope. A variety of methods have been invented and applied in imaging across the scales from macroscopic to mesoscopic to microscopic levels^1^. Optical techniques allow *in-vivo* three-dimensional imaging and interrogation of highly diffusive biological samples, ranging from image reconstruction in the macroscopic regime with fluorescence tomography^2^ further extended towards mesoscopic measurements with mesoscopic fluorescence molecular tomography^3^. For less diffusive specimens, other techniques such as light sheet microscopy^4^ and optical projection tomography^5^ are approaching the mesoscopic regime, while other such as confocal and multiphoton microscopy^6,7^ are consolidating their role for high-resolution imaging at the microscopic scale. In recent years, new advancements of imaging technology have achieved in going beyond the limits of conventional microscopy with super-resolution microscopy^8,9^, awarded a Nobel Prize in 2014. These methods heavily rely on the ability of both the hardware and the software to produce meaningful images in terms of resolution, quantification and accuracy. Especially, the tomographic imaging methods cannot abstract from the use of fast and efficient computational techniques, with experiment and algorithms being interlinked in such strong way that every improvement on one side rapidly affects the other. Furthermore, computational methods are nowadays becoming so important that they offer opportunities to develop better imaging methodologies or unlock possibilities to image under prohibitive conditions dictated by different regimes of light scattering in tissues^1^. Although tomographic principles are conceptually simple and well described and characterized, optically sectioning the sample for internal functional and structural inspection often requires a tremendous effort on both computational and experimental aspects in order to achieve a satisfying image quality.

This becomes even more relevant and demanding, in regimes at around one transport mean free path and higher, where strong light scattering restricts high-resolution imaging in the macroscopic scales. Despite the advancement in optical technologies that have pushed biological imaging beyond fundamental limits, the depth to resolution ratio still does not allow deep tissue high-resolution imaging. One solution to that problem has been provided by various and invasive chemical methods, referred to as optical clearing^10^, that chemically alter the optical properties of tissues. The price to pay in these cases is severe, since the investigated tissue needs to be fixed; thus exchanging the possibility of live *in-vivo* imaging for high resolution.

As mentioned above, Light Sheet Fluorescence Microscopy (LSFM) or Selective Plane Illumination Microscopy (SPIM) as we are going to refer to the technique throughout the text^4,11^, is one of the most widely used method for direct *in vivo*, real time^12^ visualization of internal structures and functions in model organisms. Of high scientific interest are, among others, *Caenorhabditis elegans* ^13^, *Danio rerio* (specifically in heart imaging)^14^, and multicellular tumor spheroids (MCTS)^15^. In this case, the illumination of the specimen is accomplished with a light sheet illuminating perpendicularly to the detection axis. Different camera images are recorded, either by scanning the sample by changing the position of the light sheet or translating the specimen throughout a fixed single plane illumination. In such a way, the tomographic volume is built with very little computational effort, particularly in cases of optically transparent or chemically cleared specimens. Although the technique works reasonably well in the microscopic regime, challenges arise in mesoscopy where higher scattering and absorption impede uniform and localized illumination or emission, resulting in shadows and blurring at the camera detection level. Many approaches have been proposed to tackle these complications, such as combining multiple projections at different angles^4^, pivoting the light sheet for double illumination^16^, using multi-view geometry^17^, or mixed approaches based on forward light modelling such as Mesoscopic Fluorescence Tomography (MFT)^18^. All of them require dedicated care for alignment and co-registration processes during both experimental and post-processing stages. Even the very robust and easy to implement Optical Projection Tomography (OPT)^5^, a technique based on the acquisition of projections at different angles and reconstructing the final image using back-projection algorithms, needs to take into account possible misalignments of the measuring scheme. Among other challenges^19–21^, the possibility that the sample could potentially exit the field of view along with translational and rotational misalignment drastically deteriorate the quality of the inverse reconstruction. Computational techniques for finding the Center of Rotation (CoR)^22^, correcting for sample movements^23,24^ and selecting an appropriate Region of Interest (RoI)^25^, are often used in order to overcome these shortcomings, although they are complicated and sensitive to fluctuations between measurements.

In this work, we present a new tomographic approach based on the reconstruction of the sample’s three-dimensional autocorrelation rather than on direct imaging of the specimen itself, that radically improves imaging beyond the one transport mean-free-path limit of mesoscopy. By combining the functional information from SPIM and the structural information from OPT in a complementary fashion, our new computational technique uniquely aligns the dataset by exploiting the mathematical properties of the image autocorrelation. Because of the novel use of the algorithm combined with SPIM/OPT, we found appropriate referring to this approach with the name Phase-Retrieved Tomography (PRT).

We have applied PRT for three-dimensional image reconstruction of early stage necrosis distribution in a human-breast tumor spheroid, which well represents a mesoscopic regime proof of concept scenario. MCTS is the best-characterized and most widely used scaffold-free 3D culture system that takes advantage of the inherent ability of many cancer cells to self-organize into spherical clusters^26,27^. In fact, nowadays MCTS is gaining huge attention as a pre-clinical drug-testing model and a large body of literature over the past few decades highlights the usefulness of this model system in translational cancer research and drug discovery.

In our study, we successfully reconstructed the fluorescence emitted by the spheroid’s necrotic cells in the entire volume and in resolution not accessible with SPIM/OPT alone, due to scattering and absorption, eliminating at the same time the need for data alignment and registration. By solving the phase-retrieval problem related to the three-dimensional autocorrelation, the PRT reconstruction method extends the applicability of widely used microscopy techniques towards the mesoscopic regime, providing unprecedented insights within so far turbid biological models.

## Results

### SPIM/OPT Phase-Retrieved Tomography

**Figure 1:**
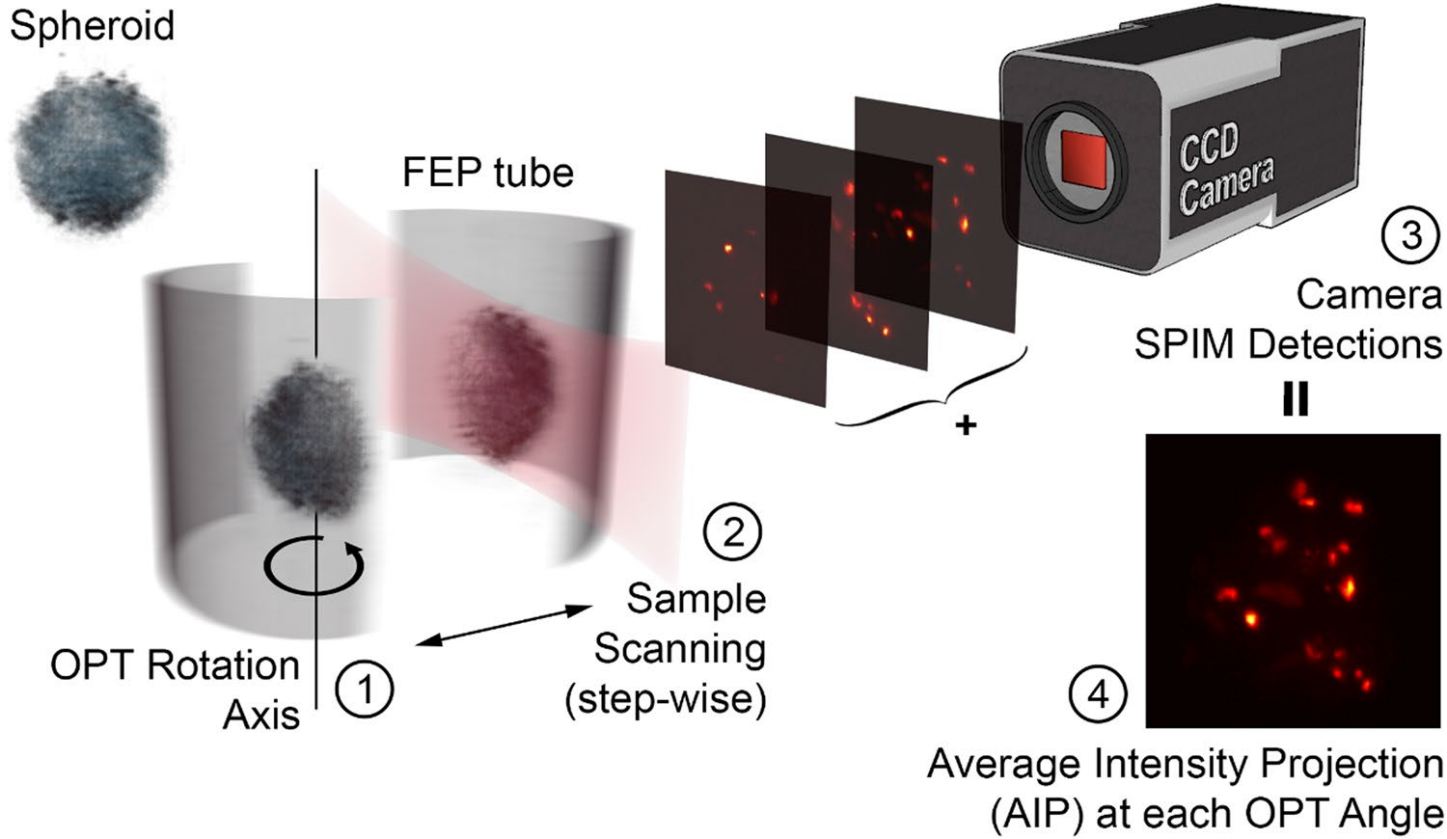
Schematic depiction of the data acquisition for the SPIM-OPT setup. Starting from the left a tumor spheroid, stained with the DRAQ7^TM^ fluorescent dye, is inserted into a FEP tube and mounted to the rotation stage. (1) The specimen is rotated at a known angle and then (2) is scanned through the light sheet along the detection axis. The illumination light sheet is established orthogonally to the detection axis and it fulfills the objective focal plane, in order to reduce out of focus contribution during the camera acquisition. While translating the specimen a SPIM detection (2D images stack) is stored (3). Finally (4), the data acquired is used to calculate the Average Intensity Projection (AIP) as a function of the angle. This procedure is repeated by rotating the sample (1) in steps of 2° until performing a complete rotation.

Employing PRT, we uniquely imaged the fluorescence distribution of the cell-death marker DRAQ7^TM^ in a T47D human breast tumor spheroid with a diameter of about 200 μm. A schematic of the experimental measurements performed in a combined SPIM/OPT setup is illustrated in Fig. 1: per each rotation angle, we acquired a collection of tomographic slices by illuminating the sample with a light sheet perpendicular to the camera plane. We placed the specimen inside a Fluorinated Ethylene Propylene (FEP) tube and we measured it over a complete rotation, making sure the whole sample was scanned during each SPIM acquisition. In case the spheroid exited the field of view while rotating, we brought it back to the center by translating along the detection focal plane (x-axis), without being concerned for such a displacement. For each SPIM dataset, at every angle of rotation, we calculated the Average Intensity Projection (AIP), stacking them one after the other as it would have happened in an OPT experiment (AIP-sinogram).

### Mesoscopic imaging of tumor spheroids

**Figure 2:**
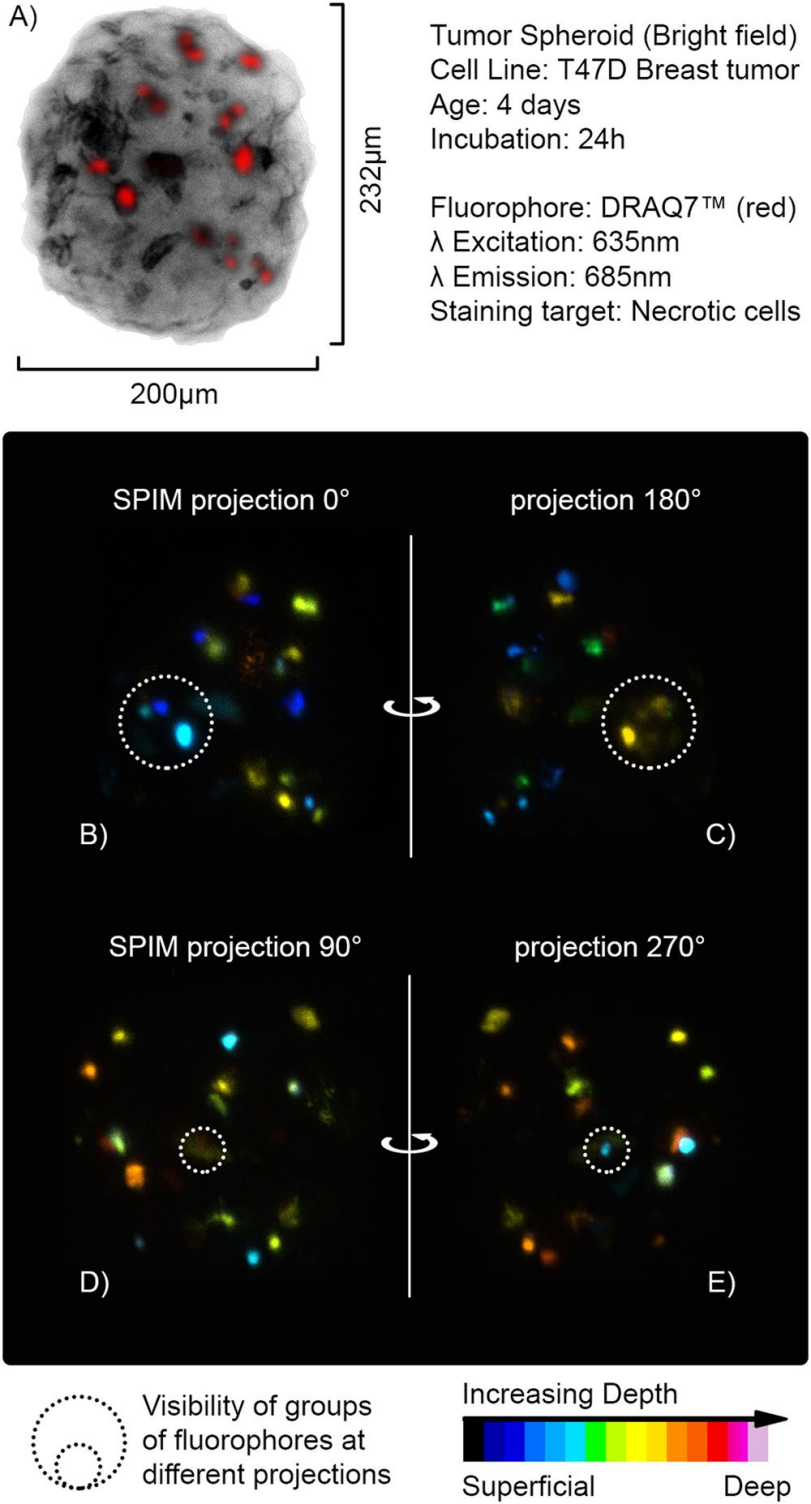
Imaging the spheroid in Mesoscopic regime. (**A**) Bright field image of the spheroid imaged at 0°. The non-uniform structure of the tumor mass is clearly noticeable. (**B–E**) SPIM-AIP at perpendicular angles with respect to one another. Dashed circles point out groups of fluorophores not visible at all measured angles, due to significant light scattering from the spheroid (mesoscopic regime).

**Figure 3:**
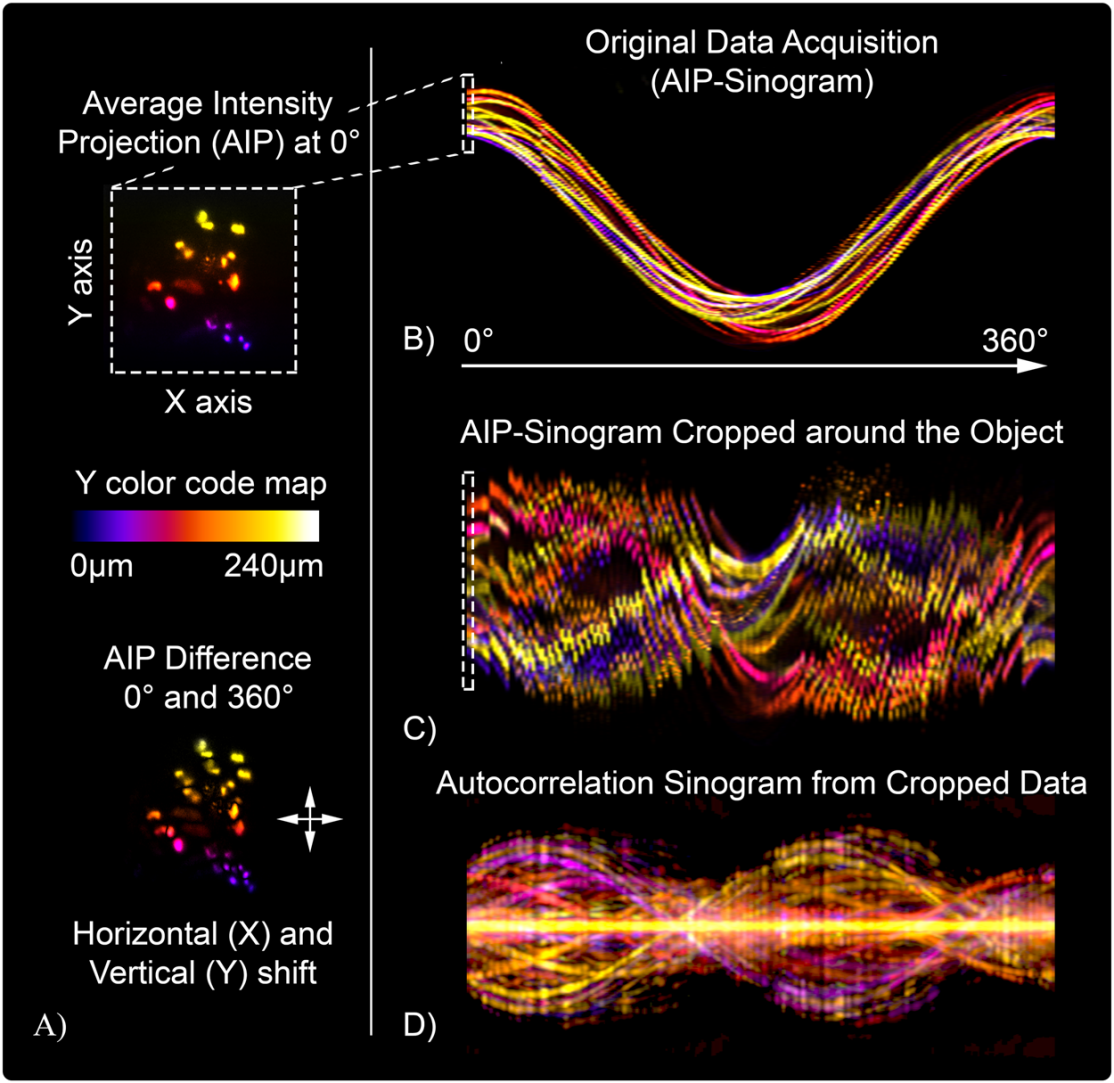
Sinogram based analysis of the acquired dataset. (**A**) AIP at 0° and relative difference after a full 360° rotation. The bidirectional drift of the sample during the measurements is visible on the bottom of panel A, and implies a misaligned AIP-sinogram. (**B**) Original sinogram of the SPIM-AIP measurements as a function of the angle, the color code that labels the Y-axis depth is the same as in panel A. It is worth noticing that the color of the sinogram turns toward blue-red at the end of the rotation, which means that the spheroid is slightly moving towards the bottom of the FEP tube. (**C**) Cropped sinogram around the spheroid used to reduce the size of the reconstructed volume. It is worth noticing how the data is completely misaligned. (**D**) Aligned autocorrelation sinogram calculated by using the data from the sinogram in (**C**). The alignment of the dataset is achieved by simply calculating the autocorrelation for each AIP projection and stacking of the projections one after the other.

**Figure 4:**
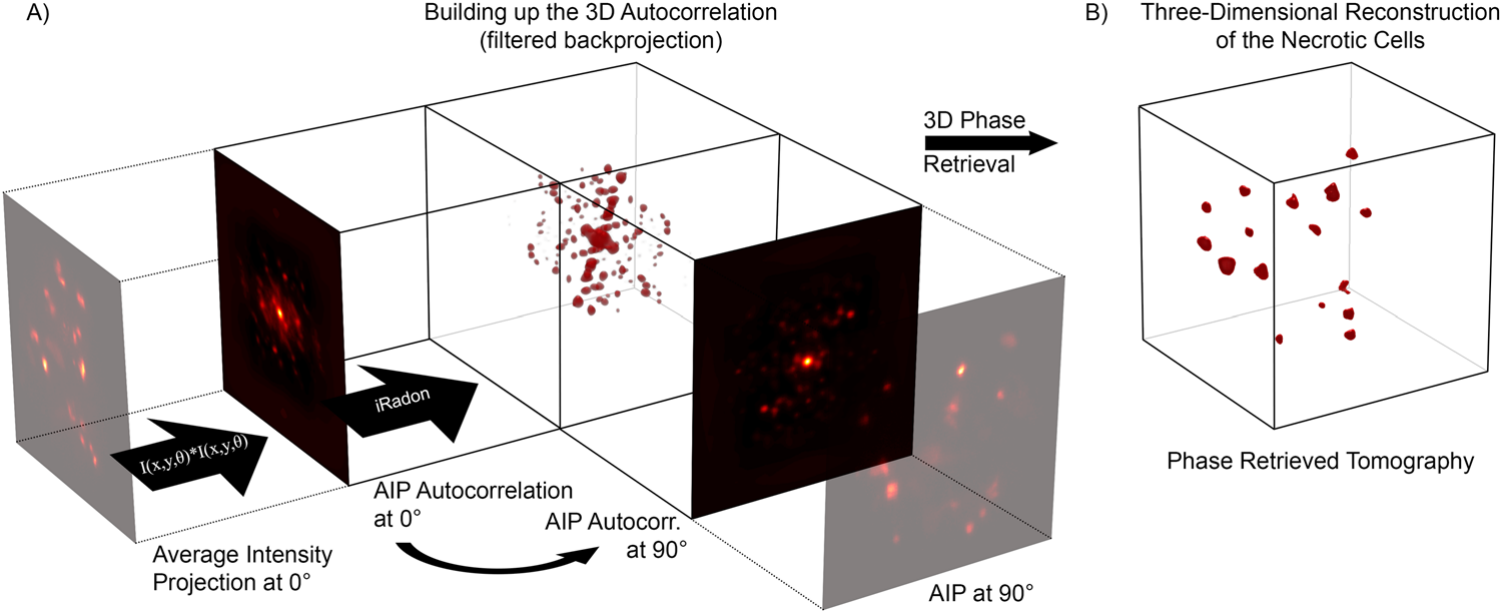
Schematics of the PRT approach. (**A**) schematic showing the backprojection criteria. For each of the Average Intensity Projections (AIP) coming from every angle (Fig. 1) we calculate the autocorrelation images. These images are smeared along a volume in function of their angle of view, following the backprojection criteria of the filtered Inverse Radon transformation. The result of this is a volume that contains the three-dimensional autocorrelation information of the object we want to image. It worth to notice that the autocorrelations are always peaked in the center and symmetric respect this point. In panel B, the final reconstruction after the Phase-Retrieved Tomography (PRT). The 3D autocorrelation feed a Gerchberg-Saxton algorithm, which retrieves the phase information reconstructing the object with no artifacts due to misalignment.

The measurements were performed in the mesoscopic regime of light scattering, as can be observed in Fig. 2. In fact, the sample is non-uniformly absorbing at visible wavelengths (bright field image in Fig. 2A) and it is large enough to scatter the fluorescent light emitted at the side opposite to the camera detection line. Indeed, in Fig. 2 (panels B–E) it is notable that groups of fluorophores are highly blurred at certain angles, while these become evident after a rotation of 180°. Therefore, in these circumstances, it is crucial to exploit all the information coming from a full rotation of the sample to accurately retrieve all hidden fluorophore distributions. Additionally, in this regime of scattering and for such a small specimen, a classical sinogram-based reconstruction approach is strongly sensitive to mechanical vibrations and off-axis rotations. The sample drift is clearly visible when comparing the projections at 0° and 360° (bottom of Fig. 3A) and yields a misaligned AIP-sinogram (Fig. 3B). With our stage, the total diagonal drift after a complete rotation was 18.7 µm, 17.6 µm along the horizontal and 6.4 µm along vertical directions in the camera image plane, which is about 10% of the whole diameter of the spheroid. We believe that the two directions of the drift were the result of the combination of inaccuracy of the rotating stage and a slow gravity-induced fall of the sample in the CyGel environment. In this difficult scenario, using simple camera projections (AIP) will turn into a misaligned sinogram, which would have to be carefully post-processed in order to correct for such displacement error. In our experiment instead, we calculated the 2D autocorrelation of each AIP, stacking them in the same order and assuming the rotation step as known. The result of this calculation is a new autocorrelation sinogram (A-sinogram) which is always perfectly centered, regardless of where the spheroid was in the camera plane (Fig. 3D). The simple backprojection of the A-sinogram, via inverse Radon transform (Supplementary Materials), leads to the calculation of a volumetric dataset corresponding to the three-dimensional autocorrelation of the sample shown in Fig. 4A. We prove this theoretically (Supplementary Materials), by showing that the autocorrelation of the projection of an object is equal to its autocorrelation projected at the same angle. Moreover, we validated numerically this assumption by calculating the 3D autocorrelation of a commonly used test-object, a three-dimensional Shepp-Logan phantom (Supplementary Materials), comparing it with what found after the backprojection of its A-sinogram. Then retrieving the phase information from such a volume, i.e. the three-dimensional autocorrelation of the real object, represents a typical phase retrieval problem with higher dimensionality^28^, here for the first time employed for tomographic purposes.

The complexity of the algorithm poses computational challenges, which are substantially related to three-dimensional Fourier transformations, and can be easily faced via parallel GPU implementation. In our specific case a normal GPU nVidia, 3-years old GeForce 780Ti with 2880 CUDA cores, could handle a volume up to 300^3^ voxels in direct space before running out of memory, a technical limitation that we plan to overcome in future algorithm developments. The result of the PRT imaging reconstruction is the volume shown in Fig. 4B, which is the indirect reconstruction of the DRAQ7^TM^ fluorescence distribution within the tumor spheroid.

### PRT validation

**Figure 5:**
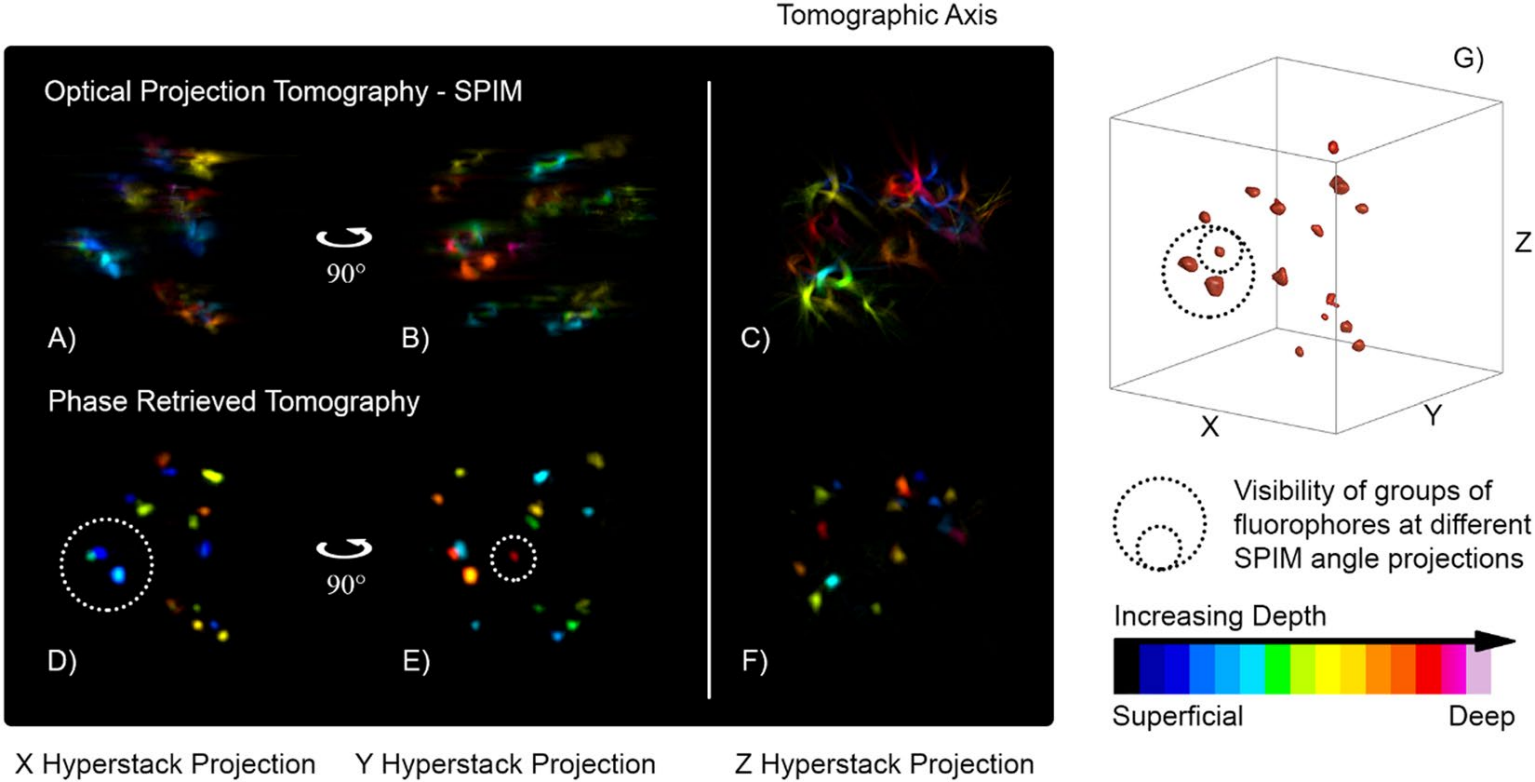
Imaging performance of the PRT technique. Hyperstack projections for the SPIM-OPT measurements (panels A–C) and the PRT reconstructions (panels D–F). The color code denotes the depth at which the fluorophore is located, starting from the first signal. It is important to notice that the two methods share the same coloring order and the same fluorophore distribution, validating our reconstruction. Moreover, PRT shows a fluorophore while SPIM cannot resolve it, due to its location on the other side of the spheroid (red to white in the color scale). The Z hyperstack suggests comparable resolutions between the two methods along the tomographic axis. Finally, panel G locates in space the groups of fluorophores dashed in panel D,E, showing that the small circle includes a cell that belongs as well to the bigger one.

To validate the PRT reconstruction we compared it with classical multi angle SPIM measurements and finally with OPT-SPIM reconstructions. First of all, we observed that the results of PRT (Fig. 5D–F) are overall consistent with some representative SPIM projections shown in Fig. 2, implying that the algorithm is correctly converging to the real fluorescence distribution. Figure 5D–F presents quantitative results for the PRT technique by visualizing the color-coded AIP (hyperstack, in which the color indicates depth) of the whole dataset (from Fig. 4B) seen from different angles. The classical Radon transform of the sinogram of Fig. 3B, co-registered by finding its CoR, leads to inaccurate reconstructions (Fig. 5A–C) in which we cannot distinguish any single necrosis, clearly demonstrating that classical approaches are not usable for this imaging regime. Similar low performance is achieved with stand-alone SPIM (Fig. 2B–E), producing blurred images for groups of fluorophores situated deep inside the spheroid (underlined by dashed circles). Instead, PRT imaging, has achieved the retrieval of highly detailed fluorescent distributions (Fig. 5D,E) with single cell resolution of ~8 μm in the three spatial dimensions (see Supplementary Material), being able to retrieve even the cells that where blurred in SPIM measurements at various projection angles (dashed circles). The correct coloring sequence of the dataset, hyper-uniform stack visualization using the Fiji toolbox^29^, efficiently displays correct distances between fluorophores compared to those of SPIM and OPT. This is a natural consequence of the correct data backprojection in the autocorrelation domain, which does not need any alignment procedures. The phase retrieval algorithm always returned comparable reconstructions, regardless of the initial and random starting guess for the phase, thus making the PRT results more efficient than the currently used techniques. Interestingly, PRT could also be used for the restoration of previously acquired datasets, which did not lead to any proper imaging due to system misalignments. We therefore believe that PRT can enter convincingly in the current biomedical imaging scene, in particular as an efficient tool for correct data reinterpretation in highly sensitive measurements.

## Discussion

We have presented, for the first time to our knowledge in biomedical imaging, the possibility to perform a three-dimensional autocorrelation reconstruction in combination with a phase retrieval algorithm to radically improve mesoscopic three-dimensional tomographic imaging of opaque biological specimens. We have validated our method on a human tumor spheroid successfully imaged in the mesoscopic regime by using a combined SPIM/OPT setup. We refer to this innovative approach as Phase-Retrieved Tomography (PRT) since it retrieves the phase related to the autocorrelation, and through the Fourier modulus, that of the entire object (See Methods Section and Supplementary Info for a detailed description and numerical validation). The new approach is insensitive to specimen translational misalignment and stage drifts in all three spatial directions, only requiring prior knowledge of the rotational degree to correctly back-project the autocorrelations sinogram. In the Supplementary Materials, we prove this theoretically and numerically, showing with a 3D Shepp-Logan phantom quantitative and robust reconstructions, even perturbing the sinogram with random shifts in two directions in the camera plane. Although the position of the retrieved object within the reconstruction volume is random, because of phase retrieval ambiguities^28^ its signal distribution is always consistent with the real object, which implies that reconstructions are not affected by data collection misalignments. As shown in Supp. Fig. 3 in comparison with the ground truth, the intensity distribution is always correctly retrieved by the PRT method, thus confirming the robustness of the reconstruction process. The experiments confirmed the same behavior and we have achieved exceptional results by collecting AIPs of full rotation in steps of 2°. Consistent reconstructions can also be achieved with bright field illumination in a classical OPT approach, as the spheroid depicted in Fig. 1 was in fact retrieved with PRT in rear bright field illumination, demonstrating the flexibility of the method with different acquisition techniques.

Of more interest are the results obtained by PRT which fully exploit the SPIM/OPT setup. To date, one of the disadvantages of SPIM is its poor resolution along the perpendicular direction with respect to the planar illumination (axial resolution). This can be improved with light sheet deconvolution^30,31^ or co-registration of stacks at different angles^32^, both of which enhance the resolution along the third direction of the reconstruction, but share the disadvantages of being highly specific to each set of measures and sensitive to drifts. On the other hand, in stand-alone OPT, accurately recovering the Center of Rotation (CoR) of the specimen is still an unsolved problem. Although many approaches have been proposed for data post-alignment^22^, they fail to provide a generalized method for removing artifacts due to misalignments. Our method manages to overcome all these issues by retrieving the object from several SPIM-AIP autocorrelations rather than direct projections, which allows the obtained autocorrelation sinogram to be inherently aligned (Fig. 3 panel D) and rotate around its center of symmetry, regardless of the original object’s rotating axis position. With our method, the need to estimate CoR or RoI from the sinograms becomes redundant, leaving the user free to focus on pure, single measurements instead of aligning the rotational system and the acquired dataset. It is worth noticing that the autocorrelation of the projection at 0° always exactly matches the one at 360° even if the sample, while rotating, does not return to the original starting position. The A-sinogram is used to compute the object three-dimensional autocorrelation, borrowing this ability from the classical OPT approach. In such a way, it is possible to also overcome the OPT weakness, i.e. the error in tracking the object while rotating, fulfilling, thus, the inverse Radon transform requirements and leading to the calculation of a nearly exact three-dimensional autocorrelation. Moreover, we found that for more sparse angular measurements (angular step greater than 2°) the PRT did not converge to correct reconstructions, due to the increased presence of star-like artifacts in the reconstructed three-dimensional autocorrelation. For more sparse reconstruction then, we believe that other approaches are needed to invert the A-sinogram, such as iterative inversion techniques like ART, SIRT, and SART^33^ that better behave with less dense angular sampling. Since they can be straightforwardly implemented for the autocorrelation reconstruction, we are going to try their usage in our following studies further improving of PRT applications.

Quantitative results are presented in Fig. 5 where the PRT reconstructions are compared with classical OPT and where the color code represents the relative depth with respect to the first fluorescent signal. The artifacts due to misalignment of the data (Fig. 5A–C) disappear in the PRT reconstruction, which convincingly demonstrates its tomographic capabilities, eliminating the need for data post-alignment (Fig. 5D–F).

SPIM hyper-stacks at four perpendicular angles as shown in Fig. 2 can also be compared to PRT reconstructions viewed from the same angles (x and y projections in the volume coordinates). The color-coded depth of each fluorophore validates the results of the PRT, which exhibits an enhanced and uniform resolution compared to that of SPIM. This is clearly demonstrated by the SPIM-AIPs underlined by dashed circles: in the mesoscopic regime, SPIM cannot resolve in all projections the presence of groups of fluorophores located on the opposite face of the spheroid, resulting in blurring and reduced non-uniform resolution. PRT, instead, clearly retrieves these objects (dash circles in Fig. 5) by fully exploiting the information coming from multiple angle AIPs and exactly combining them in autocorrelation space for enhanced depth resolution.

The novel method proposed here is beneficial in terms of correct reconstruction of relative depth and allows number estimation of the fluorophores distribution, important biological parameters to study tumor growth, cell clustering, viability and proliferation. It retrieves intensity distribution of cells with uniform resolution in three-dimension, and in addition, it is robust and easy to implement in a regular SPIM/OPT setup. Furthermore, it can be applied to the regular OPT approach using a variety of illumination schemes (bright field, fluorescence) and can also be extended to other projection based approaches, such as Xray-CT or PET. In those cases, the entire specimen would have to lie within the camera’s depth of field, otherwise defocused images would be acquired and used to calculate the autocorrelations. In our scanning approach, the plane illuminated is always in focus and its AIP is blurred only because of internal scattering, allowing, thus, better reconstructions.

Finally, our method can accept further improvements in terms of effectiveness of the Phase Retrieval algorithms used which could potentially enhance even more its accuracy and its convergence rate, pushing the community towards developing faster three-dimensional implementations. At the moment, in fact, we use the Hybrid Input-Output (HIO) retrieval method (plot of the recovery error in Supp. Fig. 4) which has been proven to converge to proper reconstructions. Other choices can be explored^28^ to efficiently treat the reconstructed 3D autocorrelation, such as sparsity-based prior information^34^ or the Oversampling Smoothness (OSS) method^35^, useful in case of noisy measurements or to attenuate backprojection artifacts. In conclusion, we point out that further validations are needed to understand the limits of the presented technique in the mesoscopic regime, possibly with the creation of specifically designed artificial samples that mimic the characteristics of the studied specimens, perhaps based on cell cultures in bioengineered scaffolds.

In the light of this, we plan to continue our work on validating and appropriately developing the PRT reconstruction technique to extend its tomographic abilities to other imaging modalities.

## Methods

### SPIM/OPT measurements

The experimental work presented here was entirely accomplished using a combined SPIM/OPT setup, in which the sample position can be software controlled along the three spatial directions and rotated along an axis perpendicular to the camera detection plane by motorized stages. The sample was imaged acquiring 180 Average Intensity Projections (AIP separated with angle step of 2°) in order to complete a full rotation. At each projection, the sample was scanned through the light sheet, having full width at half maximum of 7 μm, in steps of 20 μm while continuously recording with the camera.

For illumination, we used a continuous wave 635 nm diode laser. The light sheet was shaped by cylindrical optics and then was introduced vertically to the detection axis, having its central plane inside the focal plane of the 10x/0.28 infinity corrected detection objective (Mitutoyo, Japan). Finally, for image acquisition, a tube lens and an electron multiplying CCD (Ixon DV885, Andor Technology, Belfast, UK) were used. The camera has a resolution of 1004 × 1002 pixels and pixel size of 8 μm. The resulting pixelsize of the imaging system was 0.8 μm. With the setup described above, each SPIM scanning required an acquisition time of about 20 seconds per projection, thus leading to a total acquisition time of about one hour. This timing can be strongly reduced by further optimization of the measuring setup, but we decided to focus on the stability of the acquisition rather than the speed.

### Tumor spheroid generation

Spheroids were generated with the hanging drop method using the Perfecta3D 96-well hanging drop plates (3D Biomatrix, Ann Arbor, MI, USA) following manufacturer’s instructions. Briefly, cell suspensions for hanging drop experiments were made by dissociating cells with 0.5% trypsin-EDTA (Gibco, Grovemont Cir, Gaithersburg, MD, USA). Cell density was estimated using a hemocytometer. Dissociated cells were centrifuged at 1200 rpm for 5 min at room temperature, re-suspended in growth medium and diluted to a final concentration of 12.5 cells/µl. A 50 µl cell suspension was dispensed into each well of the spheroid culture plate to achieve an initial seeding density of 625 cells/well. In order to prevent evaporation, 1% agarose was added to the peripheral reservoirs of the hanging drop plates. The growth media was exchanged every other day by removing 25 µl of solution from a drop and replacing with 25 µl fresh media into the drop.

### Spheroid preparation for SPIM imaging

4 days old T47D breast tumor spheroids were incubated at 37 °C with 1.5 µM DRAQ7^TM^ (Biostatus, Leicestershire, UK) for 24 h prior to imaging. DRAQ7^TM^ is a far-red membrane impermeable fluorescent DNA dye that selectively stains the nuclei in dead and permeabilized cells. Staining of the spheroid with the nuclear dye was performed by replacing 10 µl from each hanging droplet with 10 µl - 5x concentrated solution of the dye. Following, spheroids were transferred from the hanging drop plate to a microscope slide, washed twice with PBS and reconstituted in 100 µl CyGel Sustain (Biostatus, Leicestershire, UK) inside a cold room (4 °C) to avoid rapid solidification of the CyGel. Then, the CyGel-embedded spheroid was transferred into a FEP tube (800 μm inner diameter, Bola, Germany) which was sealed with self-adhesive putty and then loaded on the SPIM instrument. The FEP tube containing the immobilized spheroid was embedded into a 37 °C water bath throughout the duration of the experiment to avoid liquefaction of the CyGel. The live spheroid was imaged in our custom SPIM setup using a diode laser for excitation (635 nm). The emission wavelength of the DRAQ7^TM^ fluorophore peaks at 685 nm and, accordingly a 650 nm long pass filter was used to detect the fluorescence signal.

### 3D Autocorrelation

For each SPIM dataset at different angles, we calculated the Average Intensity Projection (AIP) of every frame. The images were cropped with a squared window of 300 pixels (field of view of 240 μm) containing the whole spheroid fluorescence signal. This results in a vibrational sinogram, impossible to backproject with standard approaches (Fig. 3C). For each of the cropped AIP we calculated its autocorrelation with the Wiener-Khinchin theorem, stacking all of them in the same order. We thus obtain the autocorrelation sinogram (A-sinogram) which is always aligned. This data was backprojected with the inverse Radon transform function in MATLAB (using the default Ram-Lak filter), obtaining a cubic volume with side of 599 pixels. This volume is the three-dimensional autocorrelation of the specimen of interest. No further post processing of the data was performed.

### Phase Retrieved Tomography

The three-dimensional autocorrelation was used as an estimation of the Fourier modulus of the object to reconstruct, associating a random initial three-dimensional phase as starting point for the Phase Retrieval problem. The reconstructing window within the autocorrelation volume had the size of the object. A mixed Hybrid Input-Output (HIO) approach was used for 5000 steps followed by 1000 steps of Error-Reduction (ER). The program was implemented in MATLAB with GPU-CUDA extension and typical running time for the reconstruction was about one hour.

### Three-Dimensional Phase Retrieval algorithm

The imaging process presented is based on the calculation of autocorrelations for each camera detection at every angle of rotation. The raw camera image was cropped with a squared window of 300 pixels around the object. Then the autocorrelation $A(x,y,\theta )$ of the cropped camera image $I(x,y,\theta )$ at each step of the rotation $\theta$ is calculated accordingly to:

1 $$A(x^{\prime} ,y^{\prime} ,\theta )=I(x,y,\theta )\ast I(x,y,\theta )=\sum _{x,y}I(x,y,\theta )I(x-x^{\prime} ,y-y^{\prime} ,\theta ).$$

The $A(x^{\prime} ,y^{\prime} ,\theta )$ is the autocorrelation sinogram that we back-project with the inverse Radon transform operator (${ {\mathcal R} }^{-1})$, obtaining the three-dimensional object autocorrelation ${A}_{3D}$:

2 $${A}_{3D}(x,y,z)={ {\mathcal R} }^{-1}\{A(x^{\prime} ,y^{\prime} ,\theta )\}.$$

The autocorrelation can be related to the power spectrum $P(x,y,z)$ by the Wiener-Khinchin theorem:

3 $$P({k}_{x},{k}_{y},{k}_{z})=| {\mathcal F} \{W(x,y,z){A}_{3D}(x,y,z)\}|$$

where $W(x,y,z)$ is either a cubic or a 3D-Tukey window function that selects the core of the autocorrelation. The power spectrum can be related to the modulus of the Fourier Transform of the object $O(x,y,z)$ via the following equation:

4 $$M({k}_{x},{k}_{y},{k}_{z})=| {\mathcal F} \{O(x,y,z)\}|=\sqrt{P({k}_{x},{k}_{y},{k}_{z})}.$$

In this description $O(x,y,z)$ is the three-dimensional object that we are interested to reconstruct of which we have calculated the Fourier modulus $\,M({k}_{x},{k}_{y},{k}_{z})$. We still miss the phase information ${\rm{\Phi }}({k}_{x},{k}_{y},{k}_{z})$ which we aim to retrieve with a 3D implementation of the iterative phase retrieval algorithm described by Fienup^36^. We followed the procedure described by Bertolotti *et al*.^37^ and Katz *et al*.^38^ extending it for a three-dimensional problem solution. We describe the iteration steps according to the following numbered list. Starting from a random guess for the object ${g}_{1}(x,y,z)$ we input it in the iterative algorithm describing the k^th^ step:

5 $${G}_{k}({k}_{x},{k}_{y},{k}_{z})= {\mathcal F} \{{g}_{k}(x,y,z)\}.$$

6 $${{\rm{\Phi }}}_{{\rm{k}}}({k}_{x},{k}_{y},{k}_{z})={\rm{angle}}\{{G}_{k}({k}_{x},{k}_{y},{k}_{z})\}$$

7 $${G}_{k}\text{'}({k}_{x},{k}_{y},{k}_{z})=M({k}_{x},{k}_{y},{k}_{z}){e}^{i{{\rm{\Phi }}}_{{\rm{k}}}({k}_{x},{k}_{y},{k}_{z})}$$

8 $${g}_{k}^{^{\prime} }(x,y,z)={ {\mathcal F} }^{-1}\{{G}_{k}^{^{\prime} }({k}_{x},{k}_{y},{k}_{z})\}.$$

9 $${\rm{Constrains}}\,{\rm{of}}\,\mathrm{non} \mbox{-} \mathrm{negativity}\,{\rm{and}}\,{\rm{realness}}\,{g}_{k}^{^{\prime} }(x,y,z)\to {g}_{k+1}(x,y,z)$$

The guess ${g}_{k+1}(x,y,z)$ is calculated according to the model of the Hybrid Input-Output (HIO) algorithm for 5000 steps:

10 $${g}_{k+1}(x,y,z)=\{\begin{array}{cc}\,{g}_{k}^{^{\prime} }(x,y,z) & \mathrm{for}\,(x,y,z)\notin {\rm{\Gamma }}\\ 0\, & \mathrm{for}\,(x,y,z)\in {\rm{\Gamma }}\end{array}$$

where ${\rm{\Gamma }}$ is the region within $(x,y,z)$ that violates the constrains of non-negativity and realness and $\beta$ is a parameter that controls the convergence properties of the algorithm. In our case $\beta =0.9$ was a good choice. The entire algorithm was implemented in the Matlab environment using CUDA computation and was run on nVidia GPU. At the end of N iterations, ${g}_{N}(x,y,z)$ is the three-dimensional object reconstructed with the PRT method. In this way, we demonstrate for the first time that a three-dimensional phase retrieval algorithm is possible to be used for tomographic imaging.

## Electronic supplementary material

Supplementary information
