# Supplementary material for: Phase-Retrieved Tomography enables Mesoscopic imaging of Opaque Tumor Spheroids
Source: Sci Rep. 2017 Sep 19;7:11854. doi: 10.1038/s41598-017-12193-x (PMC5605697; doi:10.1038/s41598-017-12193-x)
Supplement: Supplementary file 1 — Supplementary information [file 41598_2017_12193_MOESM1_ESM.pdf]

Phase-Retrieved Tomography enables Mesoscopic imaging of Opaque Tumor Spheroids

Daniele Ancora[1,2*], Diego Di Battista[1,2], Georgia Giasafaki[1], Stylianos E. Psycharakis[1,3], Evangelos Liapis[1], Jorge Ripoll[4,5] and Giannis Zacharakis[1*]

[1]Institute of Electronic Structure and Laser, Foundation for Research and Technology Hellas, GR-70013 Heraklion, Greece.

[2]Department of Materials Science and Technology, University of Crete, GR-71003 Heraklion, Greece.

[3]School of Medicine, University of Crete, GR-71003 Heraklion, Greece

[4]Department of Bioengineering and Aerospace Engineering, Universidad Carlos III de Madrid, 28911 Madrid, Spain

[5]Instituto de Investigación Sanitaria del Hospital Gregorio Marañón, 28007 Madrid, Spain

*Correspondence to D.A. (email: daniele@iesl.forth.gr) or to G.Z. (email: zahari@iesl.forth.gr)

Experimental SPIM-OPT Setup:

The images presented in this work were acquired with a combined SPIM/OPT setup, shown in Supplementary Figure 1. It is composed of a custom single sided Selective Plane Illumination Microscope which is equipped with a LED illumination to perform Optical Projection Tomography as well.

For SPIM various continuous wave diode lasers are being used. In this work the output of a 635nm diode laser is used. The laser beam (colored with blue in Sup. Fig. 1) is initially expanded (BE) and then is directed to a cylindrical achromat doublet (CL) through which it is focused in a horizontal line on the corner mirror (CM). After the mirror the formed light sheet is imaged through a 2x telescope (T) to the back focal plane of the illumination objective (IO) (Mitutoyo, Plan Apo, 10x/0.28, WD=34.0mm). The telescope is placed in such a way that two conjugate planes are formed on the mirror and the back focal plane of the objective, for a better and easier adjustment of the light sheet. The formed light sheet is established orthogonally to the detection axis, intersecting with the focal plane of the detection objective.

The emitted light (colored with green in Sup. Fig. 1) is collected by a second 10x/0.28 detector objective (DO) (Plan Apo, Mitutoyo, Japan) and is projected through an apochromatic doublet tube lens (TL) (ITL200, Thorlabs) on a thermoelectrically cooled, electron multiplying CCD camera (1004x1002 pixels sensor, pixelsize: 8μm) (Ixon DV885, ANDOR Technology). Right after the objective an iris (ID) is placed in order to control the NA of the detection and thus define the depth of field and a filter wheel with appropriate fluorescence filters. For the DRAQ7 emission a 650 nm long-pass filter is used to acquire the signal.

The sample is stabilized inside a FEP tube with a solidifying agent (CyGel), and then is mounted on the sample holder which has 4 degrees of freedom. Four motorized software controlled stages allow the micrometric translation along x, y, and z-axes and rotation around the vertical y-axis. For refractive index matching, the sample is inserted inside a chamber filled with water.

In case wide field tomographic imaging is required instead of selective plane excitation and detection, the white LED Lamp is used to perform Optical Projection Tomography.

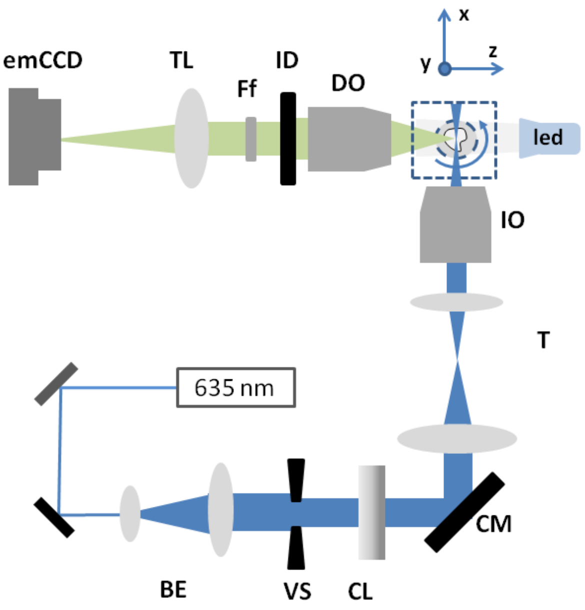

Supplementary Figure 1: The combined SPIM-OPT setup. In blue the excitation line and in green the detection axis. The sample is placed into a bath (dashed square) and translated along the z axis to perform the SPIM scanning.

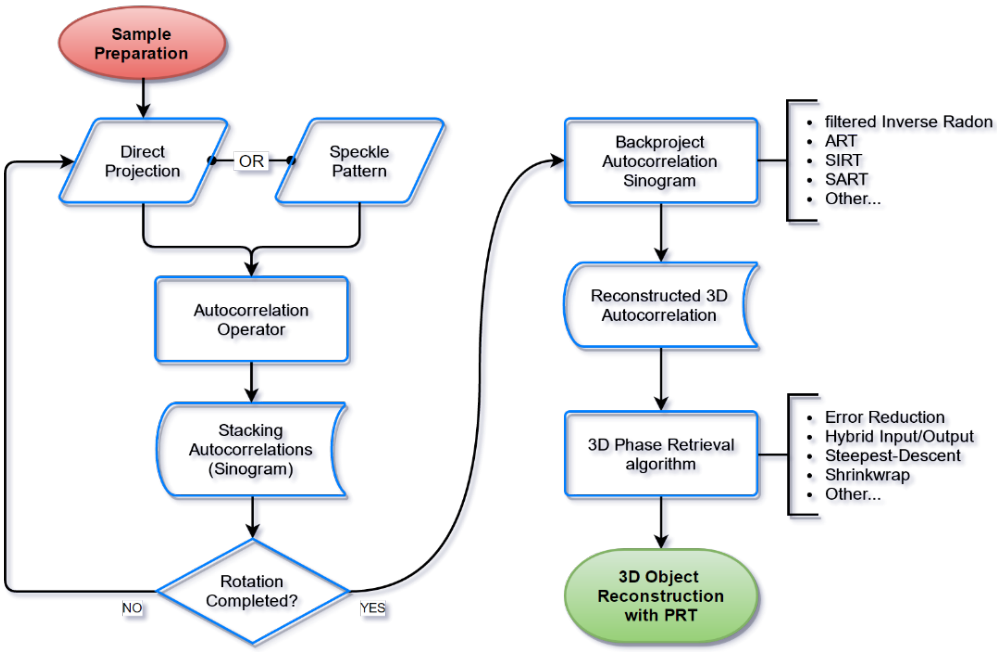

Supplementary Figure 2: Flow chart describing the Phase-Retrieved Tomography method. The process is modular and allows room for different settings, both at the acquisition and reconstruction level.

Phase-Retrieved Tomography – Flow Chart protocol

The protocol proposed for correctly reinterpreting misaligned datasets is schematically described in the flowchart of Supplementary Figure 2. The sample is placed into the setup described in the previous paragraph, and although the SPIM measurements are not strictly required, this reduces the out of focus contribution of the Average Intensity Projection (AIP). In the diagram, the Direct Projection block could be fed with different kind of projections obtained at a certain angle θ: parallel bright field projections, AIP of fluorescence excited by SPIM or simple fluorescence signal exciting the whole sample. Speckle patterns produced by a sample hidden behind a scattering curtain are also a feasible choice, since it has been proved experimentally 1, 2 and numerically 3, 4, that the camera image of both the speckle pattern and the object itself share the same autocorrelation features. Further considerations about the possibility of using speckle patterns are discussed in the last section of this document. For each acquisition, we calculate the two-dimensional autocorrelation of the unfiltered camera image, stacking them to obtain the autocorrelation sinogram. After the whole rotation is accomplished, it is possible to backproject the autocorrelation sinogram, in order to obtain the three-dimensional autocorrelation of the whole object. It is worth noticing that several techniques can be used to backproject the sinogram 5, allowing for comparative studies of different approaches. The reconstructed three-dimensional autocorrelation then is used as starting point for a phase retrieval problem, which results to the reconstruction of the object.

Phase-Retrieved Tomography – Theoretical Support

Our proposed method is based on the calculation of the three-dimensional autocorrelation of the investigated specimen and the use of a three-dimensional phase retrieval to form the final reconstruction, as shown in previous works for two dimensions 6. Calculating a 3D autocorrelation by the Radon transform of 2D camera autocorrelations requires that the autocorrelation of the projection of the specimen is equal to the projection of its three-dimensional autocorrelation at each angle. In the following section, we prove that the two quantities are identical. For simplicity, we treat the 2D case considering a two-dimensional object to be reconstructed starting from its 1D projections.

Considering (x, y) as the spatial coordinates in which the specimen exists and (η, ξ) as their respective translational coordinates, let us define the following quantities:

Object of interest: $$O(x, y) \quad (1)$$

Object autocorrelation: $$A(\eta, \xi) = O(x, y) \star O(x, y) \quad (2)$$

$$= \int O(x, y)\, O(x + \eta, y + \xi)\, dx\, dy \quad (3)$$

Projection of the Object (at angle $\theta = 0$): $$P_O(y) = \int O(x, y)\, dx \quad (4)$$

Projection of the Autocorrelation ($\theta = 0$): $$P_A(\xi) = \int A(\eta, \xi)\, d\eta \quad (5)$$

The object O is finite and limited in space, defined in a closed region $[x, y] \in \mathrm{R}$, and every integral considered is defined up to the region boundaries. In this representation $P_O(y)$ is the 1D camera detection at angle $\theta = 0$.

To be able to reconstruct the object autocorrelation by calculating the autocorrelations of the object projections literally means that the following relation must be satisfied:

$$P_O(y) \star P_O(y) = P_A(\xi). \quad (6)$$

Explicitly we can write:

$$\int P_O(y)\, P_O(y + \xi) dy = \int A(\eta, \xi)\, d\eta \quad (7)$$

$$\int P_O(y)\, P_O(y+\xi)\, dy = \int O(x,y)\, O(x+\eta, y+\xi)\, dx\, dy\, d\eta. \tag{8}$$

Both terms are integrated along y, so we can compare the arguments of the integrals:

$$P_O(y)\, P_O(y+\xi) = \int O(x,y)\, O(x+\eta, y+\xi)\, dx\, d\eta. \tag{9}$$

Now we focus on the integration along the translation η

$$P_O(y)\, P_O(y+\xi) = \int O(x,y) \left\{ \int O(x+\eta, y+\xi)\, d\eta \right\} dx. \tag{10}$$

It is possible however, to make the following consideration regarding the argument in brackets; for finite objects in space, a linear translation does not influence their definite integral, i.e. the projection is preserved for translations along the axis perpendicular to the detection axis. This means that integrating in the translation η, *projects* the object into its perpendicular axis, eliminating the dependence in x (one coordinate could be seen as the translation of the other) and hence:

$$\int O(x+\eta, y+\xi)\, d\eta = \int O(x+\eta, y+\xi)\, dx = P_O(y+\xi). \tag{11}$$

This implies that:

$$\begin{aligned} P_O(y)\, P_O(y+\xi) &= \int O(x,y)\, P_O(y+\xi)\, dx \\ &= P_O(y+\xi) \int O(x,y)\, dx \\ &= P_O(y+\xi)\, P_O(y). \end{aligned} \tag{12}$$

Which exactly proves our initial claim. Theoretically then, it is possible to calculate the autocorrelation of the object by the Inverse Radon transformation of the autocorrelation of the projections.

Phase-Retrieved Tomography – Numerical Validation

To test the validity of the proposed method, we performed a numerical validation using a three dimensional Shepp-Logan phantom, a commonly used model for testing the performance of reconstruction algorithms. We used a freely available tool for the generation of such phantom in the Matlab environment 7, creating a cubic volume of 128 pixels per side. The whole process is shown in Supplementary Figure 3, in which each image is the Average Intensity Projection (AIP) along the axis of rotation. The 3D phantom was Radon transformed in the domain of $\theta \in [0°, 180°]$ with steps separated by $1°$ using the corresponding Matlab function. This results in the direct projections sinogram, which is perfectly aligned since the axis of rotation around which we performed the Radon transform was fixed to the center of the image. We verified that the reconstruction obtained inverting the sinogram with the filtered inverse Radon transform gives correct results (Supplementary Figure 3C). We then simulated a vibrational perturbation in the sample rotation by displacing it randomly in three directions at each θ of the radon transform, resulting in a misaligned projections' sinogram shown in panel D. The random shift introduced was in the range of [-5, +5] pixels in every spatial direction. In this case, the calculation of the inverse Radon transform results to a noisy reconstruction (panel E), in which features are no longer visible. Vibrational misalignment and the subsequent reconstruction artifacts represent an unsolved problem in real experiments and appropriate reconstruction algorithms have not been demonstrated to date. Here we introduce a solution based on the calculation of the autocorrelation for each of the projections, in such a way that we create an autocorrelation sinogram which is perfectly aligned, as we have recently presented in one of our preliminary studies 3. We noticed a perfect agreement between the three-dimensional autocorrelation calculated from the direct object and the one reconstructed from the sinogram (Supplementary Figure 3 panels F-H). In practice, the quality of the 3D autocorrelation is never affected by the vibrational-noise level in the original sinogram, in fact any displacement is simply not propagated in the autocorrelation space. Since we have a better estimation for the three-dimensional object's autocorrelation rather than

the object itself, we can exploit this information with a three-dimensional implementation of a phase retrieval algorithm, in order to accomplish the reconstruction and retrieve the real object. As can be seen in panel I, the reconstruction obtained with Phase-Retrieved Tomography (PRT) matches the original object (panel A) even if the dataset was perturbed with strong vibrational noise.

Finally, to monitor the convergence of the reconstruction it is possible to calculate the recovery error, defined by the Euclidean distance between the autocorrelation and the autocorrelation of the retrieved object at each step (Supplementary Figure 4). In an ideal case, when the phase is fully retrieved, this quantity is minimized and the two autocorrelations converge. It is possible to notice that already after 5000 steps of HIO the solution is already stable, while the eventual addition of 1000 steps of Error Reduction (ER) 6 does not alter significantly the results. In any case, we prefer to do not use ER steps due to the fact that the algorithm might force sharpness in the reconstructed volume.

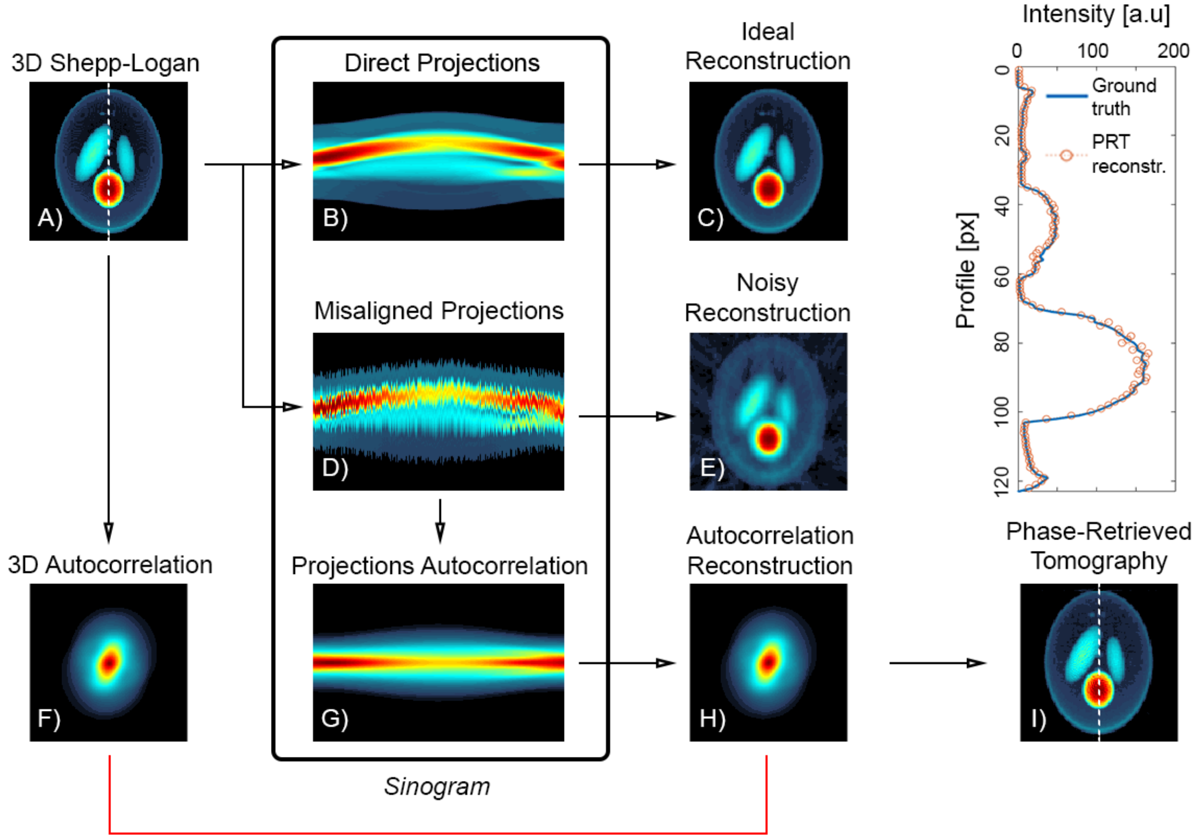

Supplementary Figure 3: A) 3D Shepp-Logan phantom used to test the reconstruction algorithm in comparison with classical backprojection methods. B) Sinogram of the projections at various angles, which is obtained as the Radon transform of the object in the range $\theta \in [0°, 180°]$ and C) its inverse radon transform. D) Misaligned measurements due to random shift of the object while rotating, that perturbs the sinogram and return wrong reconstruction at E). F) Original object's autocorrelation directly calculated. G) Naturally aligned sinogram of the autocorrelation of each single projection from D) and its inverse radon transform H). It is worth noticing the agreement between F) and H). Finally, I) is the Phase-Retrieved Tomography reconstruction of the object using only the backprojected H). PRT correctly retrieves intensity information as proven in the graph in the upper right corner of the figure, showing the intensity profiles of both panels A) and I).

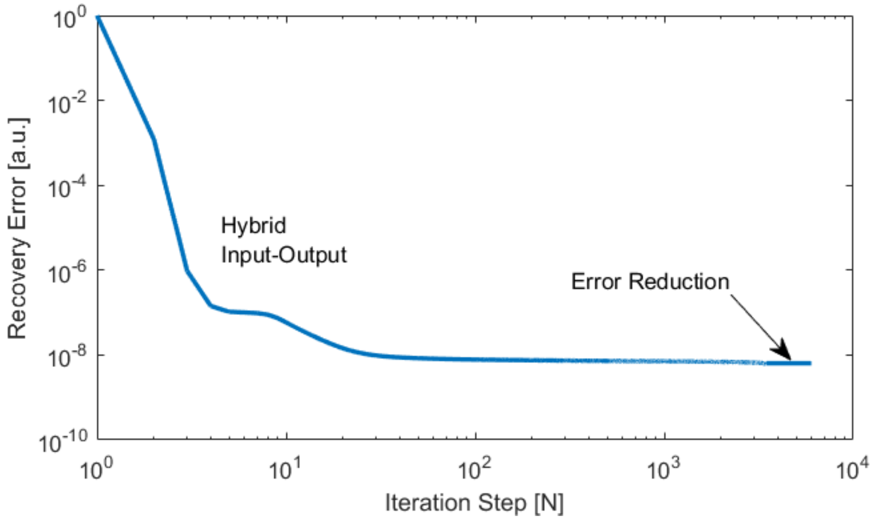

Supplementary Figure 4: Recovery error in function of the minimization step plotted in log-log scale. We can notice how after a fast minimization, the solution converges towards a stable plateau already with the HIO iterations. Following ER steps do not further modify the retrieved solution.

Phase-Retrieved Tomography – Resolution and Cross sections

In this section we present the results obtained with PRT in comparison to that of normal OPT-SPIM reconstructions. The results of classical OPT-SPIM reconstruction, corrected for the not centered axis of rotation, lead to a misaligned recontruction affected by rotational noise (Supp. Fig. 5 panel A, left image). Such circular artifacts, in this case, visibly affect the results, inhibiting a clear determination of the fluorophore distribution. With PRT instead (Supp. Fig. 5 panel A, right image), each single fluorescent target results visible and the overall result is not influenced by the sample misalignment. By looking directly at cross sections of the reconstructed volume, the gain in resolution is even more evident (Supp. Fig. 5 panel B-C): with classical reconstruction (left column) a single cell is reconstructed having an annular shape typical of misaligned measurement, while with PRT (right column) it is possible to locate the single cells shown in each slice with the corresponding top view of the whole volume reconstruction. This suggests that we have achieved single cell resolution also in the tomographic axis, being limited by the optical imaging setup itself. In the specific case treated in this work, the final resolution obtained with PRT resulted to be equal to approximately $\sim 10\ px = 8\ \mu m$ in the three spatial dimension. Lastly, we notice that such resolutions seem to be not affected by the discretization procedure implied by the usage of multiple Discrete Fourier Transorms (DFT), since we reconstructed always smooth and rounded objects as expected when imaging fluorescing cells.

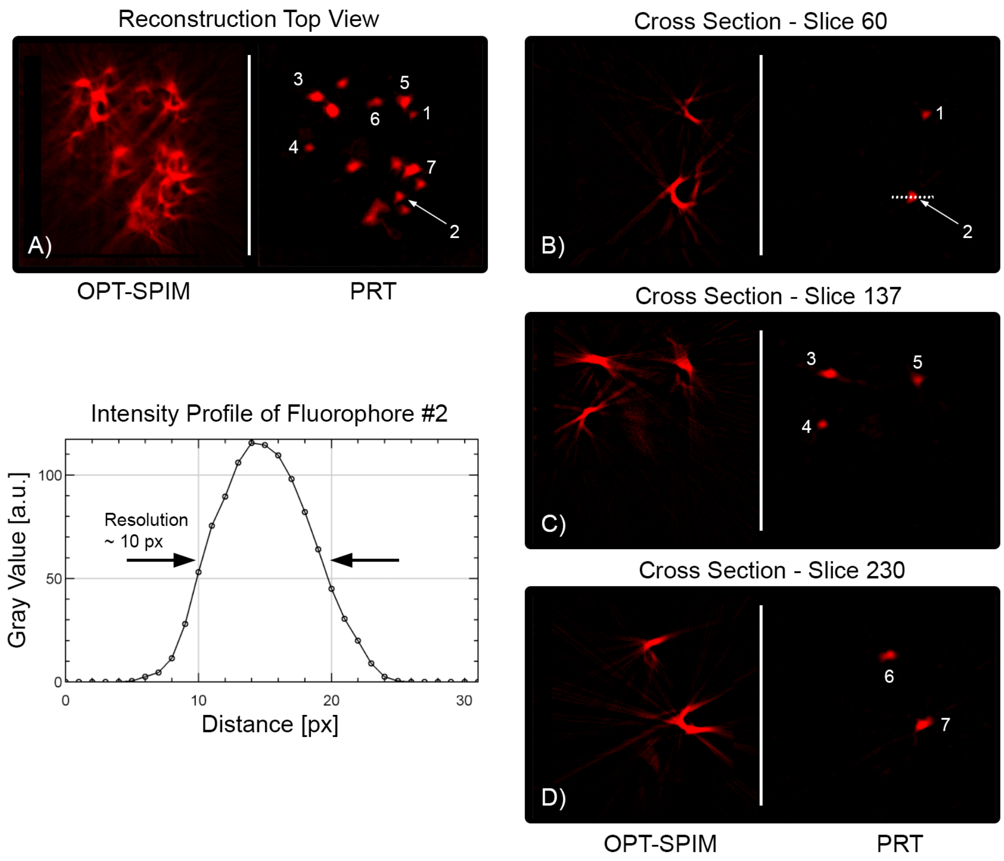

Supplementary Figure 5: Analysis of the reconstructed volume with classical and PRT approaches. A) Top view (average intensity projection) of the tomographic axis for the volumes reconstructed with the different methodologies. B-D) Cross-sections of the volume and their corresponding location with respect to A). The plot in the bottom-left part of the figure shows the single-cell resolution obtained with PRT reconstruction along the tomographic axis.

Phase-Retrieved Tomography – Discussion on its applicability for hidden 3D imaging

A radical new approach in overcoming the limitations imposed by multiple light scattering in biological media, is based on the use of active optical elements for the accurate control of the impinging wavefront, which provides a compensation for the random refractive index variations in tissue. Counterintuitively then, multiple scattering in the optical paths can be exploited to predetermine light propagation and to focus through or inside turbid biological media 8 9. These pioneering approaches are based on the utilization of adaptive dynamic wavefront shaping techniques applied for controlling and inversing light diffusion, thus leading to the ability of focusing in or through highly scattering media. In this context, a growing number of works presents clever solutions to achieve structured illumination behind highly diffusive media, achieving non-diffractive 10 or more interestingly light sheet focusing 11 behind scattering curtains.

On the other hand, new techniques based on autocorrelation imaging are currently being employed in applications that allow, under certain conditions dictated by the memory effect range (intrinsic isoplanatism) 12, the visualization of objects hidden behind biological turbid media or behind corners 1 13. Optical Imaging in such extreme conditions has been performed only in two-dimensions by exploiting the speckle autocorrelation property which, within the memory effect 12 regime, is identical to the autocorrelation of the object itself. The conceptual idea behind these studies is based on the fact that the autocorrelation of the signal is preserved while being scrambled by the scattering media within a certain range. The so-produced speckle retains the autocorrelation properties of the object and it relates to it through the Fourier Transform (FT) modulus. The autocorrelation of the speckle produced in front of the turbid layer can feed a Gerchberg–Saxton algorithm 14 used to retrieve its Fourier phase, allowing the reconstruction of the hidden object. Although mathematically the phase retrieval process works particularly well at every dimensionality 15, except for the lack of uniqueness in 1D problems, for optical imaging purposes (to the best of our knowledge) it has been used only in 2D implementations.

In this scenario, it is worth taking into account another key aspect of this work. Because of its design, the PRT protocol can potentially be implemented for imaging hidden three-dimensional specimens behind scattering curtains or around corners. In principle, in fact, PRT can tackle the current lack of techniques for high-resolution 3D imaging of hidden objects by exploiting speckle pattern resulting from the object positioned at different angles rather than single projections. The speckle pattern sequence generated by a specimen fluorescing behind a curtain that encloses it still contain the autocorrelation information needed to correctly perform the reconstruction. Preliminary numerical studies 3 4, in fact, already shown the possibility to correctly calculate the autocorrelation sinogram of a three-dimensional object hidden behind a random phase scrambling media. Such an A-sinogram, is identical to the A-sinogram of the object itself and can be processed via PRT methods to correctly retrieve the hidden three-dimensional object. We are already facing the challenges of a 3D hidden imaging reconstruction and currently we are on the process of testing it in experimental measurements.

References.

1. Bertolotti, J. *et al.*, Non-invasive imaging through opaque scattering layers. *Nature* **491** (7423), 232-234 (2012).
2. Katz, O., Heidmann, P., Fink, M. & Gigan, S., Non-invasive single-shot imaging through scattering layers and around corners via speckle correlations. *Nat. Photon.* **8**, 784–790 (2014).
3. Ancora, D. *et al.*, Phase-retrieved optical projection tomography for 3D imaging through scattering layers. *Proc. of SPIE* **9718**, 97181B (2016).
4. Ancora, D. *et al.*, Optical projection tomography via phase retrieval algorithms for hidden three dimensional imaging. *Proc. of SPIE* **10074**, 100741E (2017).

5. Beister, M., Kolditz, D. & Kalender, W. A., Iterative reconstruction methods in X-ray CT. *Phys. Medica* **28** (2), 94-108 (2012).

6. Shechtman, Y. *et al.*, Phase Retrieval with Application to Optical Imaging. *IEEE Signal Process. Mag.* **32** (3), 87-109 (2015).

7. Shabel, M., 3D Shepp-Logan phantom, Available at https://www.mathworks.com/matlabcentral/fileexchange/9416-3d-shepp-logan-phantom/content/phantom3d.m (2006).

8. Mosk, A. P., Lagendijk, A., Lerosey, G. & Fink, M., Controlling waves in space and time for imaging and focusing in complex. *Nat. Photon.* **6** (5), 283-292 (2012).

9. Gigan, S., Optical microscopy aims deep. *Nat. Photon.* **11** (1), 14-16 (2017).

10. Di Battista, D., Ancora, D., Leonetti, M. & Zacharakis, G., Tailoring non-diffractive beams from amorphous light speckles. *Appl. Phys. Lett.* **109** (12), 121110 (2016).

11. Di Battista, D. *et al.*, Tailored light sheets through opaque cylindrical lenses. *Optica* **3** (11), 1237-1240 (2016).

12. Freund, I., Rosenbluh, M. & Feng, S., Memory effects in propagation of optical waves through disordered media. *Phys. Rev. Lett.* **61** (20), 2328-2331 (1988).

13. Katz, O., Heidmann, P., Fink, M. & Gigan, S., Non-invasive single-shot imaging through scattering layers and around corners via speckle correlations. *Nat. Photon.* **8**, 784–790 (2014).

14. Fienup, J. R., Phase retrieval algorithms: a personal tour [Invited]. *Appl. Opt.* **52** (1), 45-56 (2013).

15. Shechtman, Y. *et al.*, Phase Retrieval with Application to Optical Imaging. *IEEE Signal Process. Mag.* **32** (3), 87-109 (2015).
